# Abnormal Ipsilesional Hemifields and Improvement Post-Biofeedback Training in Patients with Hemianopia Measured with the MAIA Microperimeter

**DOI:** 10.3390/brainsci16070759

**Published:** 2026-07-20

**Authors:** Jessica Cao, Ghaliah Nsour, Katrina Manas, Zainab Farooq, Samuel N. Markowitz, Monica Daibert-Nido

**Affiliations:** 1 Low Vision Program, Department of Ophthalmology, Toronto Western Hospital, 399 Bathurst St., 6th Floor, Room 620, Toronto, ON M5T 2S8, Canada; jessica.cao@mail.utoronto.ca (J.C.); ghaliah.nsour@mail.utoronto.ca (G.N.); ktmanas@up.edu.ph (K.M.); snm1@rogers.com (S.N.M.); 2 Department of Ophthalmology and Vision Sciences, Faculty of Medicine, University of Toronto, 340 College Street, Suite 400, Toronto, ON M5T 3A9, Canada; 3 Saint Kateri Tekakwitha Catholic Secondary School, 221 Fundy Bay Boulevard, Vaughan, ON L4K 1S7, Canada; 4 Krembil Research Institute, 60 Leonard Avenue, Toronto, ON M5T 2S8, Canada; 5 Donald K. Johnson Eye Institute, Krembil Discovery Tower, 8th Floor, Neurology, 399 Bathurst Street, Toronto, ON M5T 2S8, Canada

**Keywords:** stroke, brain tumor, biofeedback training, retinal sensitivity, MAIA microperimetry, sightblindness, brain trauma, brain infection

## Abstract

**Introduction**: This study analyzes the ipsilesional hemifields in patients with hemianopia and biofeedback training (BT). **Methods**: Prospectively, BT stimulated the paracentral ipsilesional hemifields. BT was compared to a control group using C 10-2 program 68 points on the MAIA microperimetry (Centervue, Padova, Italy). Five weekly BT sessions of 20 min were delivered. A total of 34 subjects enrolled, with 28 in treatment, and six in control group. Outcomes: General retinal sensitivity (RS), average of the central two columns, the paracentral column of the blind hemifield, ipsilesional hemifield, and the whole ipsilesional and blind hemifields. Paired *t*-tests were used for statistical analysis. **Results**: Control and treatment groups were equal demographically. In the control group, no difference was found pre-and-post BT. Baseline RS on the ipsilesional hemifield was 21.4 ± 4.5, lower than the normal cut-off value of 25 dB (*p* < 0.001). BT improved RS from 14.0 ± 4.2 to 15.0 ± 4.8, *p* = 0.01, paracentral two columns, 16.3 ± 5.3 to 17.8 ± 5.6, *p* = 0.01, central column in ipsilesional hemifield, 20.7 ± 5.4 to 21.8 ± 4.8, *p* = 0.05, and ipsilesional hemifield, 21.42 ± 4.8 to 22.39 ± 4.24), *p* = 0.01. **Conclusions**: Ipsilesional hemifields were abnormal and BT improved RS, benefiting patients with hemianopia.

## 1. Introduction

Homonymous hemianopia (HH) is one of the most disabling forms of visual impairment and the most common visual deficit following lesions of the retro-chiasmal visual pathways. Most commonly resulting from stroke, traumatic brain injury, brain tumors, or neurosurgical procedures, HH affects approximately 30–50% of individuals with post-chiasmal cerebral injury. Stroke remains the leading cause, with approximately 70% of posterior cerebral artery infarcts resulting in visual field loss [1]. Although spontaneous recovery may occur during the early post-injury period, persistent visual field defects are common, and effective rehabilitation strategies remain an important unmet clinical need.

The loss of one hemifield significantly impairs visual search, reading, navigation, obstacle avoidance, driving eligibility, and overall functional independence [2,3,4,5,6]. Patients with HH exhibit abnormal saccades that impair visual scanning, spatial exploration, and orientation [2]. Consequently, mobility, independent ambulation, and quality of life are substantially reduced [3,4]. Reading impairment is another common complaint, largely due to parafoveal visual field references loss and disrupted fixation control [5]. In addition, visual field loss alters the perceived subjective visual midline, contributing to impaired balance and an increased risk of falls [6].

Traditionally, the ipsilesional visual hemifield has been considered functionally intact in HH. However, emerging evidence suggests that subtle functional abnormalities may also be present within these supposedly unaffected hemifields, a phenomenon referred to as sightblindness [7]. In contrast to blindsight, which has been extensively investigated in patients with HH, the functional integrity of the central visual field and ipsilesional hemifield has received comparatively little attention despite its relevance for visual rehabilitation. Recent studies show that neither the central visual field nor the ipsilesional hemifield is entirely normal in patients with HH [8].

The method used to assess visual fields may influence the detection of these subtle deficits. Unlike conventional automated perimetry, microperimetry combines retinal imaging with real-time eye tracking, allowing the precise correlation between retinal location and visual sensitivity. A recent study comparing the MAIA microperimeter with the Humphrey Field Analyzer in patients with HH demonstrated that microperimetry may detect more subtle visual field abnormalities, likely owing to its continuous retinal tracking and greater fixation accuracy [9].

Although observation remains the standard approach for many patients with HH, some rehabilitation centers employ prism adaptation and compensatory saccadic training to improve visual function [10,11,12,13]. More recently, evidence has suggested that visual perceptual training may partially restore visual fields in cortical blindness while also reducing transsynaptic retinal ganglion cell degeneration in the corresponding affected hemiretina [14,15,16,17].

Our group has been investigating an emerging vision rehabilitation technique known as microperimetry-guided biofeedback training (BT) for patients with HH. Originally developed more than two decades ago for central vision loss by training eccentric fixation [18], BT provides real-time audio-luminous feedback based on retinal fixation, promoting fixation stability while enhancing visual attention through alternative neural pathways. BT showed increasing visual cortical activity in patients with Stargardt’s disease [19].

More recently, we applied this technique to patients with hemianopia and demonstrated significant improvements in visual function and vision-related self-referred quality of life [20]. In that study, BT consisted of fixation training using bimodal audio-luminous stimuli projected to a retinal locus located two to three degrees within the ipsilesional hemifield, adjacent to the transition zone near the fovea. Twelve treated patients demonstrated significant improvements in visual function and self-reported quality of life.

The present prospective non-randomized controlled study aimed to (1) determine whether the ipsilesional hemifield demonstrates abnormalities on microperimetry in patients with HH; and (2) evaluate whether microperimetry-guided biofeedback training can improve retinal sensitivity within this supposedly unaffected hemifield.

## 2. Methods

This was a prospective, interventional, non-randomized, controlled study. Patients with various stable causes of brain injury and a diagnosis of hemianopia confirmed through MRI and visual fields were included. The 20 central degrees of the visual fields were assessed using the Macular Integrity Assessment (MAIA) microperimeter (Centerview, Padova, Italy), C 10-2 program, 4-2 strategy.

Patients were recruited from the Low Vision Clinic at Toronto Western Hospital, University of Toronto, Canada. Inclusion criteria were as follows: (1) a diagnosis of homonymous hemianopia confirmed by visual field testing; (2) brain injury of any etiologies; (3) age between 18 and 90 years; and (4) the ability to understand and follow the visual and auditory stimuli as well as the training instructions.

Exclusion criteria included previous perceptual learning or vision rehabilitation training for hemianopia, significant ocular pathology, and cognitive impairment that would preclude reliable testing or participation in the training protocol.

The control group consisted of patients who were able and willing to attend the baseline visit and a follow-up assessment 6 weeks later without receiving the intervention. Randomization was not feasible because of logistical constraints during the study period, including major hospital construction that substantially limited access to parking and the hospital facilities. Consequently, control participants were selected based on their availability and willingness to return for the follow-up visit despite not receiving treatment. This resulted in unequal group sizes and may have introduced selection bias. Randomized allocation will be implemented in a subsequent study.

**Figure 1:**
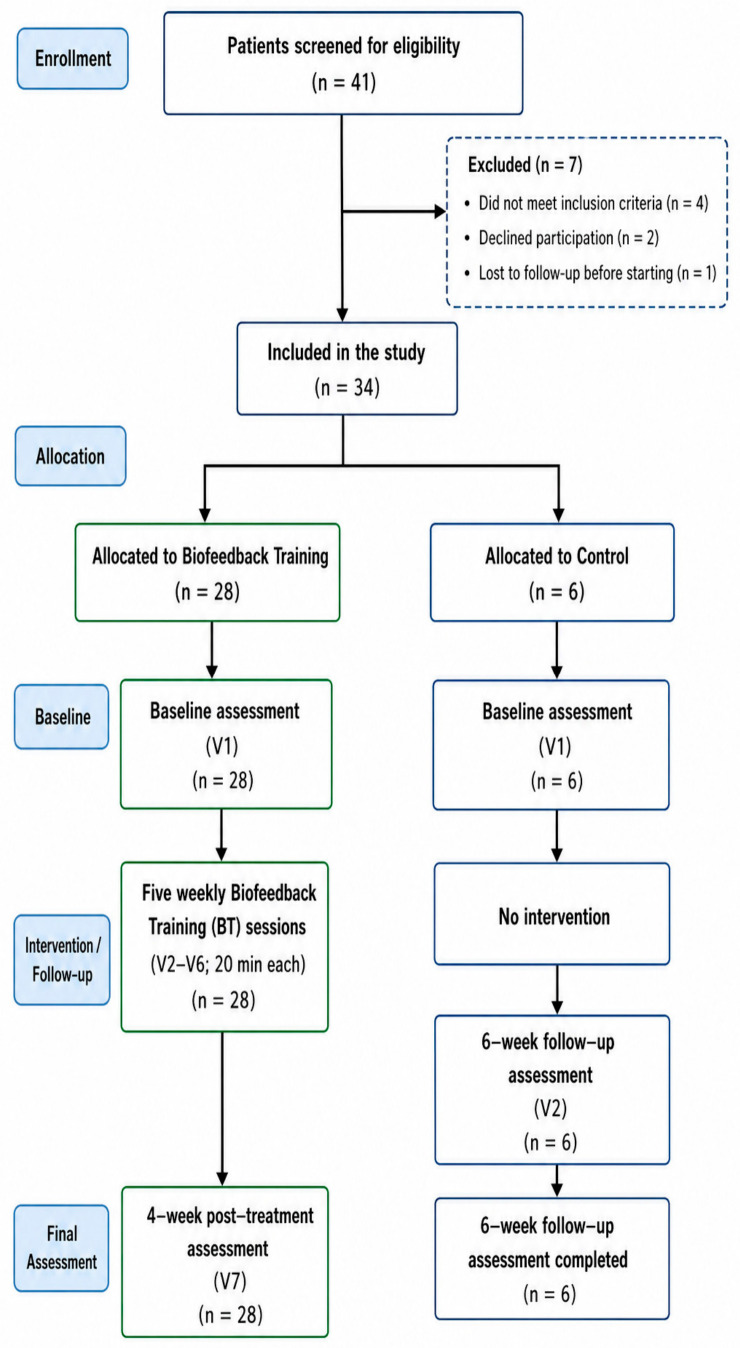
Clinical trial flowchart. CONSORT-style flow diagram of participant recruitment and follow-up. Forty-one patients with homonymous hemianopia were screened for eligibility. Thirty-four participants met the inclusion criteria and were enrolled in the study. The patients were allocated in control and treatment groups. After a baseline visit, twenty-eight participants underwent the intervention. Five weekly sessions of microperimetry-guided biofeedback training followed by a follow up for post-treatment assessment 4 weeks after baseline, while six participants served as controls and underwent repeat assessment after 6 weeks.

Patients had one baseline visit 1 (V1), five BT visits (V2–6), and 1 month follow-up visit (V7). The control group had one baseline visit (V1) and one control visit (V2) 6 weeks later that assessed the same variables as the baseline visit (Figure 1).

### 2.1. Ethics Statement

The study was approved by the University Health Network Research Ethics Board, reference number 20-5618 (Toronto, ON, Canada) and registered at ClinicalTrials.gov (NCT05397873A). Data was collected from July 2021 to November 2022. Written informed consent was obtained from all patients.

### 2.2. Assessment and Outcomes

For the treated and control groups, during V1, an MAIA microperimeter test was performed using the C 10-2, 4-2 strategy, 68 stimuli program. For the microperimetry and fixation tests, a small red circle with 0.76 degrees was used for fixation.

Participants underwent a structured audio-luminous biofeedback training (BT) program to stimulate areas of residual function within the paracentral ipsilesional visual field described elsewhere [20]. Five BT weekly sessions of 20 min each (100 min in total) were delivered.

**Figure 2:**
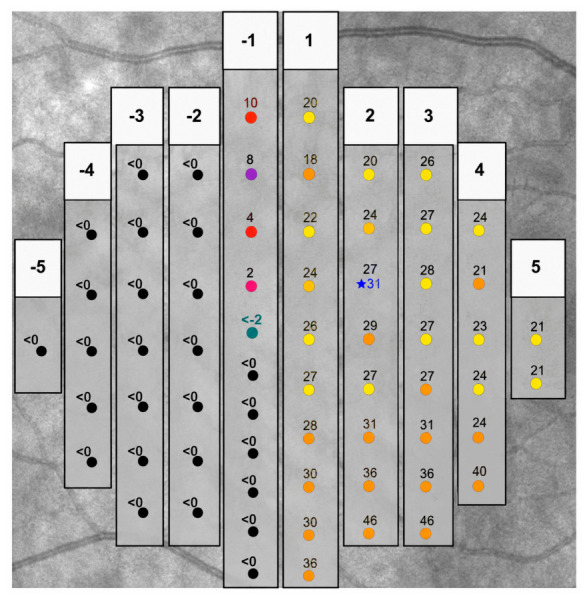
MAIA Microperimetry C 10-2 68 point program for the OD of a patient in the study: paracentral retinal sensitivity (PRS, average of the retinal sensitivity 20 points in the paracentral 2 columns, −1 and 1), average retinal sensitivity in the central column of the blind hemifield (−1), average retinal sensitivity in the central column of the ipsilesional hemifield (1), average retinal sensitivity in the ipsilesional hemifield (1–5), and average retinal sensitivity in the blind hemifield (−1 to −5).

The primary outcome was the Average Retinal Sensitivity of the 68 points measured by the MAIA (whole-field RS). Secondary outcomes were (Figure 2) paracentral retinal sensitivity (PRS, average of the retinal sensitivity 20 points in the paracentral 2 columns, −1 to 1), average retinal sensitivity in the central column of the blind hemifield (−1), average retinal sensitivity in the central column of the ipsilesional hemifield (1), average retinal sensitivity in the ipsiesional hemifield (1–5), and average retinal sensitivity in the blind hemifield (−1 to −5), as shown in Figure 2.

In order to compare the clinical characteristics between groups, distance vision, near vision, reading speed and fixation stability were also collected on V1.

### 2.3. Intervention

**Figure 3:**
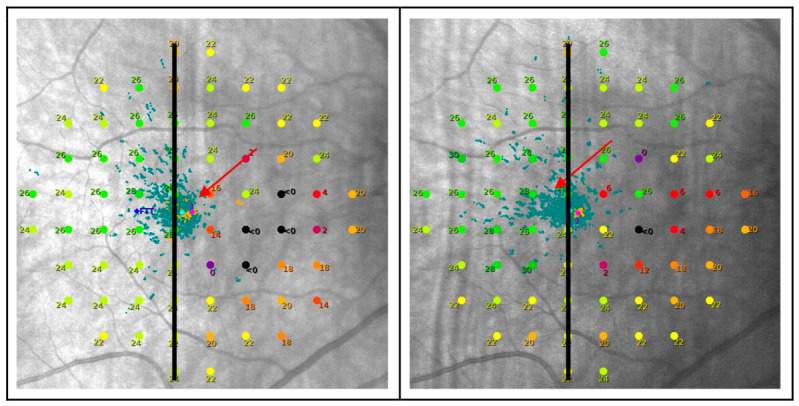
Pre-and-Post BT fixation on the MAIA microperimetry C10-2, right eye. (**Left**) Each green point is an attempt of fixation. Preferred retinal locus (PRL) center are located initially between 16 and 28 dB point (red arrows). The FTT (in blue) is the fixation training target. (**Right**) fixation was relocated effectively 1.5 degree to the left, that is, ipsilesional hemifield, between the 26 and 26dB points, followed by an improvement of retinal sensitivity.

Audio-luminous BT was performed on the MAIA microperimeter (Centervue, Padova, Italy), using the biofeedback module. On top of the microperimetry C10-2 test done on the same device, the trained retinal locus (TRL) was determined. This locus should be located between 2 and 3° horizontally from the fovea, and toward the seeing hemifield on the retina, or blind hemi-visual field, bringing the fixation to a larger sensitivity area on the retina. The eye ipsilesional to the hemianopia was treated. Both eyes remained open during the procedure. Only the treated eye was stimulated. The training technique was previously described in our study [20]. Ideally, after BT sessions, the patient would fixate using the TRL for reading, seeing faces, and others, voluntarily or unconsciously (Figure 3).

From V2 to V6, BT was performed weekly.

### 2.4. Statistical Analysis

Data were summarized using descriptive statistics. The distribution of continuous variables was assessed using the Shapiro–Wilk test. Because the pre- and post-treatment retinal sensitivity data were not normally distributed, within-group comparisons were performed using the Wilcoxon signed-rank test. Baseline continuous variables between the treatment and control groups were compared using Student’s *t*-test, whereas categorical variables were compared using Fisher’s exact test or the χ^2^ test, as appropriate. A two-sided *p* value < 0.05 was considered statistically significant.

All participants had complete pre- and post-treatment microperimetry data. Occasional missing values that did not occur would be excluded from the corresponding analyses.

## 3. Results

A total of 34 patients was studied in total. A total of 28 patients was in the treated group and six patients were in the control group. There was no difference between the control and treatment groups in terms of sex, age, treated eye, or diagnosis (see Table 1). The demographic characteristics and time after the brain injury are described in Table 2.

Comparing the treated and control groups, no difference was found in the clinical characteristics (Table 3).

Statistical analysis: Baseline values were compared between the treatment and control groups using an unpaired Student’s *t*-test. No statistically significant differences were observed between groups for any of the evaluated outcome measures (all *p* > 0.05).

**Figure 4:**
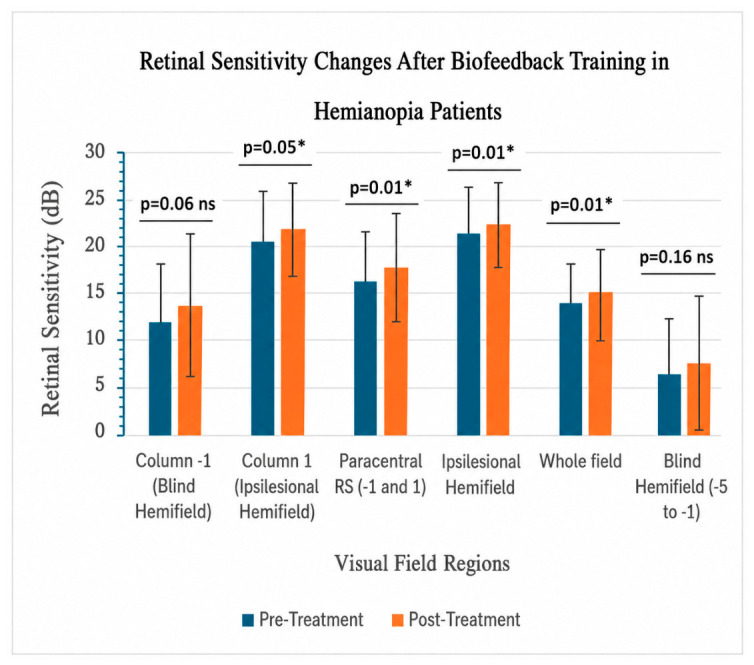
Comparison of the retinal sensitivity within the different regions of the fields between pre-and post-BT. * means statistical significance. Columns and 1 in the ipsilesional hemifield improved the retinal sensitivity. In the blind hemifield, paracentral retinal sensitivity improved and column. −1 showed a trend for improvement. The whole ipsilesional hemifield improved overall.

**Figure 5:**
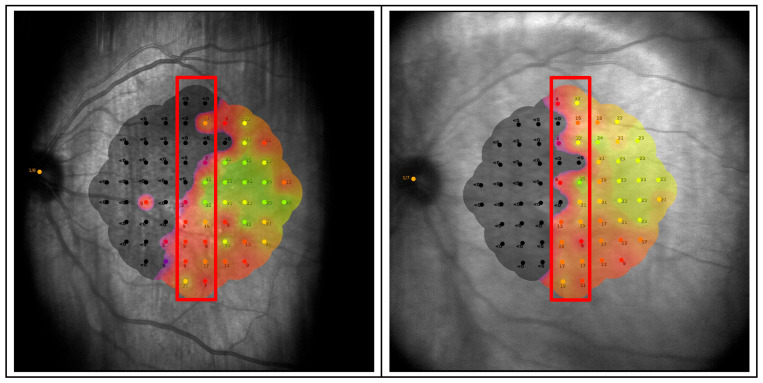
**Increase in retinal sensitivity for one case.** Microperimetric results of a patient who improved her retinal sensitivity in the columns +1 and −1 post-BT (inside the red box). On the left, C 10-2 MAIA microperimetry Pre-BT, Retinal Sensitivity 9 dB, paracentral columns 20 points average (Paracentral Retinal Sensitivity) 8.65 dB. On the right are the results for post-BT, retinal sensitivity 10.1 dB, and paracentral retinal sensitivity 11.9 dB. There was a 3.25 dB increase in the paracentral area. The image refers to a single participant.

Table 4 and Figure 4 show that the treated group had significant improvements post-BT. For the average retinal sensitivity of the 68 points measured (whole hemifield RS), there was an improvement from 14 ± 4.2 dB to 15 ± 4.8 dB (*p* = 0.01). The paracentral retinal sensitivity (PRS, average of the retinal sensitivity 20 points in the paracentral 2 columns,-1 to 1) improved from 16.3 ± 5.3 dB to 17.8 ± 5.6 dB (*p* = 0.01), and the ipsilesional hemifield (1 to 5) improved from 21.42 ± 4.8 to 22.3 ± 4.2 dB (*p* = 0.01). The average retinal sensitivity in the central column of the blind hemifield (-1) showed a trend for improvement, from 11.9 ± 6.4 to 13.8 ± 7.6 (*p* = 0.06), and the average retinal sensitivity in the central column of the ipsilesional hemifield (1) improved from 20.7 ± 5.4 to 21.8 ± 4.8 (*p* = 0.05). The blind hemifield did not improve in the treated group. An illustration of this improvement is shown in Figure 5.

Noticeably, as shown in Table 5, regarding the retinal sensitivity on the ipsilesional hemifields, considered in general to be the “sound hemifields”, the currently accepted cut-off for a normal retinal sensitivity for the MAIA microperimeter is of 25 dB [21]. Before BT, the mean pre-treatment RS on the ipsilesional hemifield for all patients (control plus treated) was 21.4 ± 4.5, significantly lower than the normal cut-off value of 25 dB (*p* < 0.001).

## 4. Discussion

Our study found that the mean retinal sensitivity in the ipsilesional hemifield (columns 1–5) of patients with homonymous hemianopia was approximately 4 dB lower than the age-matched normative values provided by the MAIA microperimeter [20], although limitations exist regarding the use of a universal normal cut-off. This difference between the baseline values and the normative values exceeds the reported test–retest variability of MAIA microperimetry. In patients with age-related macular degeneration (AMD), the 95% limits of agreement for mean retinal sensitivity are approximately 4.2 dB when all test points are analyzed and improve to 3.2 dB when only abnormal points are included, suggesting that changes exceeding these values are unlikely to reflect measurement variability alone [22]. Since the retinal locations analyzed in the present study were supposedly considered abnormal, the observed reduction likely would represent a functional impairment. Although baseline retinal sensitivity was reduced by 4 dB compared to normative values, the magnitude of improvement observed after training (approximately 1–1.5 dB) lies within the reported test–retest variability of MAIA microperimetry. As such, our findings on biofeedback training should be interpreted cautiously and require confirmation in larger controlled studies.

Previous studies have demonstrated that patients with hemianopia exhibit diminished spatial and temporal sensitivities within their apparently intact hemifield [8,9,21], including reduced contrast sensitivity, prolonged reaction times, and task-dependent visual deficits. These findings suggest that the ipsilesional hemifield may not function entirely normally and one of the reasons could be the impaired integration of visual information from the damaged hemisphere. Using the Useful Field of View (UFOV) test, Karlijn et al. identified visual impairments affecting activities such as driving and object recognition that were not detected by conventional perimetry, highlighting subtle sensory deficits in the “intact” hemifield [8]. Similarly, the MAIA microperimeter has demonstrated greater sensitivity than standard automated perimetry for detecting functional abnormalities in patients with homonymous hemianopia because of differences in stimulus characteristics, background luminance, and intensity range [10].

Given the importance of the residual ipsilesional hemifield for activities of daily living, its rehabilitation may represent an additional therapeutic target alongside the training of blind visual fields. Although the improvements observed after biofeedback training were modest (approximately 1–1.5 dB depending on retinal location), their clinical significance is a possibility because a minimal clinically important difference has not been established for MAIA retinal sensitivity in homonymous hemianopia. Our previous pilot study encountered a 2.7 ± 0.9 dB increase in columns −1 to 1 after BT in 9/11 participants, also using the MAIA [20], and Amore et al. had a 1.9 ± 0.3 dB increase using the MP1 [23]. However, these findings should be considered preliminary evidence of improved retinal sensitivity rather than definitive proof of clinically meaningful functional benefit.

Computational models of the adult visual cortex suggest that repeated visual stimulation may strengthen existing synaptic connections and promote the functional reorganization of neighboring neural networks [24]. One possible explanation for the observed improvements is the Residual Vision Activation Theory, which proposes that neuroplasticity occurs primarily within areas of residual vision or relative visual field defects [25]. According to this hypothesis, retinal locations bordering the transition between seeing and non-seeing visual fields—approximately corresponding to columns −2, −1, 1, and 2 in the present study—may retain partially preserved neural networks that remain responsive to repeated stimulation. Training a single retinal locus may therefore activate adjacent networks and reinforce neuronal connections beyond the trained area. Although this mechanism provides a biologically plausible explanation for our findings, no neuroimaging or electrophysiological assessments were performed in the present study, and these proposed mechanisms remain speculative.

Repeated exposure to microperimetry may improve performance through familiarity with the examination rather than physiological recovery. However, the absence of significant retinal sensitivity changes across all retinal sectors in the control group argues against a major practice effect, although the small number of control participants limits this conclusion. Likewise, retinal sensitivity analyses were performed without adjustment for multiple comparisons because the study was exploratory and the evaluated retinal regions were anatomically related rather than independent endpoints. Nevertheless, this increases the possibility of type I error, particularly for findings with borderline statistical significance such as the central ipsilesional column (*p* = 0.05). Accordingly, these results should be regarded as hypothesis-generating and require confirmation in adequately powered randomized studies.

Consistent with previous reports, no significant improvements were observed within the blind hemifield, although a trend toward improvement was noted in the central border region (column −1), aligned with the Residual Vision Activation Theory.

Previous studies have consistently demonstrated that MAIA- and MP1/3-based audio-luminous biofeedback improves visual acuity, fixation stability, retinal sensitivity, reading speed, and quality of life in patients with central vision loss and nystagmus [26,27,28,29,30]. In retinal diseases, biofeedback training has also been associated with cortical reorganization, whereby cortical representations shift from the damaged fovea to a newly established preferred retinal locus [31]. Similarly, combining intravitreal dexamethasone with biofeedback has been shown to improve best-corrected visual acuity, retinal sensitivity, fixation stability, and macular thickness more than pharmacological treatment alone, with the authors proposing that enhanced fixation stability promotes communication between the retina and visual cortex and facilitates cortical plasticity [32]. Functional MRI studies in Stargardt disease further demonstrated increased activation of the primary visual cortex following biofeedback training, supporting this hypothesis [19]. Whether similar mechanisms occur in hemianopia remains unknown because neuroimaging was not performed in the present study. Future functional MRI studies may clarify the neural mechanisms underlying biofeedback-induced improvements.

This study has several limitations. The prospective but non-randomized design and the marked imbalance between treatment and control groups increase the possibility of selection bias and limit causal inference. The small control group reduced statistical power to detect spontaneous fluctuations and the practice effects associated with repeated microperimetry. Multiple retinal sensitivity outcomes were analyzed without adjustment for multiple comparisons, increasing the possibility of type I error. Furthermore, because of the very small and unbalanced control group, direct statistical comparisons of change scores between the treatment and control groups were not performed. Future randomized studies with adequate sample sizes should incorporate between-group analyses, such as change-score comparisons or mixed-effects repeated-measures models, to provide a more robust estimate of the treatment effect. In addition, patient-centered outcomes such as reading performance, visual search, mobility, and vision-related quality of life were not evaluated. Finally, because no neurophysiological or neuroimaging measures were obtained, the proposed neuroplastic mechanisms remain biologically plausible hypotheses rather than direct evidence. Larger randomized studies incorporating functional MRI are warranted to confirm these findings and further elucidate the mechanisms and therapeutic potential of biofeedback training in homonymous hemianopia.

## 5. Conclusions

The present study demonstrates that the ipsilesional hemifield in patients with homonymous hemianopia exhibits reduced retinal sensitivity compared to normative MAIA values, suggesting that this supposedly unaffected hemifield is functionally impaired. Microperimetry-guided biofeedback training was associated with modest improvements in retinal sensitivity within the trained retinal regions, supporting the potential of this rehabilitation strategy to enhance residual visual function. Although the observed improvements should be interpreted cautiously because of exploratory design and methodological limitations, these findings contribute to the growing evidence that the ipsilesional hemifield represents a potential target for visual rehabilitation. Larger randomized controlled trials incorporating functional outcome measures and neuroimaging are needed to determine the clinical relevance and underlying mechanisms of these observations.

## Figures and Tables

**Table 1:** Baseline demographic and clinical characteristics of the study participants.

Characteristic	Control (n = 6)	Biofeedback Training (n = 28)	*p* Value
Sex			0.67
Female	2 (33.3%)	12 (42.9%)	
Male	4 (66.7%)	16 (57.1%)	
Age (years), mean ± SD			0.52
	52.3 ± 28.7	60.6 ± 15.0	
Treated eye			0.91
Right	2 (33.3%)	10 (35.7%)	
Left	4 (66.7%)	18 (64.3%)	
Cause of hemianopia			0.20
Stroke	5 (83.3%)	22 (78.6%)	
Neurosurgery/tumor	1 (16.7%)	5 (17.9%)	
Herpes encephalitis	0	1 (3.6%)	

Data are presented as mean ± standard deviation (SD) or number (%). *p* values compare baseline characteristics between the control and biofeedback training groups using the appropriate statistical tests (Student’s *t*-test for continuous variables and Fisher’s exact test or χ^2^ test for categorical variables, as appropriate).

**Table 2:** Clinical characteristics of the study participants.

ID	Age (years)	Sex	Ethnicity	Etiology	Treated Eye	Time Since Brain Injury (months)
Biofeedback Training Group (n = 28)						
1	57	M	Black	Stroke	L	7
2	40	F	Caucasian	Stroke	L	12
3	72	M	Caucasian	Herpes encephalitis	L	7
4	64	F	Caucasian	Neurosurgery/Tumor	R	6
5	82	M	Latin	Stroke	R	26
9	80	F	Asian	Stroke	L	7
10	63	M	Asian	Neurosurgery/Tumor	R	14
11	84	F	Caucasian	Stroke	L	12
12	51	F	Caucasian	Stroke	R	10
14	57	M	Asian	Stroke	L	5
15	62	M	Caucasian	Stroke	L	3
16	80	F	Caucasian	Stroke	L	8
19	65	F	Caucasian	Stroke	R	15
18	68	F	Black	Stroke	L	5
20	64	F	Caucasian	Stroke	L	12
21	64	F	Asian	Stroke	L	8
22	58	F	Caucasian	Stroke	R	6
23	48	M	Caucasian	Stroke	L	6
25	51	F	Caucasian	Stroke	R	6
26	34	M	Asian	Stroke	R	10
27	17	M	Caucasian	Neurosurgery/Tumor	L	19
28	69	M	Caucasian	Stroke	L	5
30	47	M	Asian	Stroke	L	24
31	74	M	Caucasian	Stroke	L	18
32	18	M	Asian	Neurosurgery/Oncology	L	12
33	42	M	Caucasian	Stroke	R	7
36	55	M	Asian	Neurosurgery/Tumor	L	6
37	57	M	Black	Stroke	R	11
Control Group (n = 6)						
38	17	M	Black	Neurosurgery/Tumor	L	13
39	39	F	Caucasian	Stroke	R	14
40	90	F	Latin	Stroke	R	6
41	61	M	Caucasian	Stroke	L	7
42	78	M	Caucasian	Stroke	L	6
43	29	M	Caucasian	Stroke	L	8

Abbreviations: F = female; M = male; L = left; R = right.

**Table 3:** Baseline comparison of visual function parameters between the treatment and control groups.

Outcome Measure	*p* Value
Near visual acuity	0.219
Distance visual acuity (OD)	0.207
Distance visual acuity (OS)	0.300
Reading speed	0.757
Whole-field retinal sensitivity	0.623
Paracentral retinal sensitivity	0.237
Fixation stability	0.709

Abbreviations: OD = right eye; OS = left eye. Baseline values compared using an unpaired Student’s *t*-test.

**Table 4:** Retinal sensitivity measured by MAIA microperimetry before and after biofeedback training.

Outcome (dB)	Baseline Mean ± SD (95% CI)	Post-BT Mean ± SD (95% CI)	*p* Value
Control Group (n = 6)			
Whole field (RS)	13.9 ± 3.4 (9.8–19.2)	13.9 ± 3.7 (9.9–19.7)	0.92
Blind hemifield (−5 to −1)	6.3 ± 3.5 (1.0–12.0)	6.3 ± 4.3 (1.5–12.3)	0.92
Ipsilesional hemifield (1 to 5)	21.4 ± 3.1 (17.7–26.5)	21.6 ± 2.6 (18.1–25.3)	0.60
Paracentral field (−1 to 1)	15.0 ± 3.5 (10.6–20.0)	15.8 ± 3.2 (11.2–19.4)	0.35
Central column (−1)	9.8 ± 4.8 (3.0–16.1)	10.5 ± 4.2 (5.2–14.9)	0.35
Central column (1)	20.1 ± 3.6 (16.0–26.4)	21.1 ± 3.2 (17.2–25.8)	0.35
Biofeedback Training Group (n = 28)			
Whole field (RS)	14.0 ± 4.2 (1.6–20.9)	15.0 ± 4.8 (3.8–27.5)	0.01
Blind hemifield (−5 to −1)	6.5 ± 5.9 (0.2–19.7)	7.6 ± 7.2 (0.0–25.6)	0.16
Ipsilesional hemifield (1 to 5)	21.4 ± 4.8 (2.5–28.5)	22.4 ± 4.2 (7.5–29.4)	0.01
Paracentral field (−1 to 1)	16.3 ± 5.3 (2.2–24.5)	17.8 ± 5.6 (3.8–30.7)	0.01
Central column (−1)	11.9 ± 6.4 (0.3–23.0)	13.8 ± 7.6 (0.0–31.1)	0.06
Central column (1)	20.7 ± 5.4 (2.2–29.1)	21.8 ± 4.8 (7.3–30.7)	0.05

Abbreviations: RS = retinal sensitivity; BT = biofeedback training; CI = confidence interval. Values are presented as mean ± SD (95% CI). *p* values were calculated using the Wilcoxon signed-rank test. Significant *p* values (<0.05) are shown in bold in the original table.

**Table 5:** Baseline retinal sensitivity in the ipsilesional (“Sound”) hemifield compared to the MAIA normative reference value.

Variable	Mean ± SD (dB)	Reference Value (dB)	*p* Value
Baseline ipsilesional (“sound”) hemifield retinal sensitivity (all participants, n = 34)	21.4 ± 4.5	25.0	<0.001

Data represent baseline measurements from all participants (control and biofeedback training groups combined). The normative retinal sensitivity value of 25 dB was obtained from published MAIA reference data.

## Data Availability

The data presented in this study are available from the corresponding author upon reasonable request. The data are not publicly available due to privacy and ethical restrictions.

## References

[1] Wolberg A., Tripathy K., Kapoor N. (2024). Homonymous Hemianopsia. StatPearls [Internet].

[2] Gao Y., Sabel B.A. (2017). Microsaccade dysfunction and adaptation in hemianopia after stroke. Restor. Neurol. Neurosci..

[3] Goodwin D. (2014). Homonymous hemianopia: Challenges and solutions. Clin. Ophthalmol..

[4] Warren M. (2009). Pilot study on activities of daily living limitations in adults with hemianopsia. Am. J. Occup. Ther..

[5] Chokron S., Perez C., Peyrin C. (2016). Behavioral consequences and cortical reorganization in homonymous hemianopia. Front. Syst. Neurosci..

[6] Leff A.P., Scott S.K., Crewes H., Hodgson T.L., Cowey A., Howard D. (2000). Impaired reading in patients with right hemianopia. Ann. Neurol..

[7] Wing J.J., Burke J.F., Clarke P.J., Feng C., Skolarus L.E. (2017). The role of the environment in falls among stroke survivors. Arch. Gerontol. Geriatr..

[8] Cavézian C., Perez C., Peyrin C., Gaudry I., Obadia M., Gout O., Chokron S. (2015). Hemisphere-dependent ipsilesional deficits in hemianopia: Sightblindness in the ‘intact’ visual field. Cortex.

[9] Bola M., Gall C., Sabel B.A. (2013). “Sightblind”: Perceptual deficits in the “intact” visual field. Front. Neurol..

[10] Peli E. (2000). Field expansion for homonymous hemianopia by optically induced peripheral exotropia. Optom. Vis. Sci..

[11] Sahraie A., Smania N., Zihl J. (2016). Use of NeuroEyeCoach to improve eye movement efficacy in patients with homonymous visual field loss. Biomed. Res. Int..

[12] Weinberg J., Diller L., Gordon W.A., Gerstman L.J., Lieberman A. (1977). Visual scanning training effect on reading-related tasks in acquired right brain damage. Arch. Phys. Med. Rehabil..

[13] Pollock A., Hazelton C., Rowe F.J., Jonuscheit S., Kernohan A., Angilley J., Henderson C.A., Langhorne P., Livingstone K., Dhillon B. (2019). Interventions for visual field defects in people with stroke. Cochrane Database Syst. Rev..

[14] Fahrenthold B.K., Cavanaugh M.R., Tamhankar M.A., Lam B.L., Feldon S.E., Johnson B.A., Redmond B.V., Yang J., Saionz E.L., Huxlin K.R. (2024). Training in cortically blinded fields appears to confer patient-specific benefit against retinal thinning. Investig. Ophthalmol. Vis. Sci..

[15] Saionz E.L., Busza A., Huxlin K.R., Levin L.A. (2022). Chapter 25—Rehabilitation of visual perception in cortical blindness. Handbook of Clinical Neurology.

[16] Mueller I., Poggel D., Kenkel S., Kasten E., Sabel B.A. (2003). Vision restoration therapy after brain damage: Subjective improvements of activities of daily life and their relationship to visual field enlargements. Vis. Impair. Res..

[17] Qian T., Xu X., Liu X., Yen M., Zhou H., Mao M., Wang X., Xu X. (2022). Efficacy of MP-3 microperimeter biofeedback fixation training for low vision rehabilitation in patients with maculopathy. BMC Ophthalmol..

[18] Melillo P., Prinster A., Di Iorio V., Olivo G., D’Alterio F.M., Cocozza S., Testa F., Simonelli F., Brunetti A. (2020). Biofeedback rehabilitation and visual cortex response in Stargardt’s disease: A randomized controlled trial. Transl. Vis. Sci. Technol..

[19] Misawa M., Pyatova Y., Sen A., Markowitz M., Markowitz S.N., Reber M., Daibert-Nido M. (2023). Innovative vision rehabilitation method for hemianopsia: Comparing pre- and post-audio-luminous biofeedback training for ocular motility, improving visual functions and quality of life. Front. Neurol..

[20] Charng J., Sanfilippo P.G., Attia M.S., Dolliver M., Arunachalam S., Chew A.L., Wong E.N., Mackey D.A., Chen F.K. (2020). Interpreting MAIA microperimetry using age- and retinal loci-specific reference thresholds. Transl. Vis. Sci. Technol..

[21] Woutersen K., Geuzebroek A.C., van den Berg A.V., Goossens J. (2020). Useful field of view performance in the intact visual field of hemianopia patients. Investig. Ophthalmol. Vis. Sci..

[22] Barkana Y., Pondorfer S.G., Schmitz-Valckenberg S., Russ H., Finger R.P. (2021). Improved sensitivity of microperimetric outcomes for clinical studies in age-related macular degeneration. Sci. Rep..

[23] de Rossi F., Guidobaldi M., Moscato U., Marra C., Amore F. (2020). Rehabilitative effect of the patterned stimulation with MP1 microperimeter in homonymous hemianopia. Vis. Rehabil. Int..

[24] Andrade M.A., Muró E.M., Morán F. (2001). Simulation of plasticity in the adult visual cortex. Biol. Cybern..

[25] Sabel B.A., Henrich-Noack P., Fedorov A., Gall C. (2011). Vision restoration after brain and retina damage: The “Residual Vision Activation Theory”. Prog. Brain Res..

[26] Amore F.M., Paliotta S., Silvestri V., Piscopo P., Turco S., Reibaldi A. (2013). Biofeedback stimulation in patients with age-related macular degeneration: Comparison between two different methods. Can. J. Ophthalmol..

[27] Morales M.U., Saker S., Wilde C., Rubinstein M., Limoli P., Amoaku W.M. (2020). Biofeedback fixation training method for improving eccentric vision in patients with loss of foveal function secondary to different maculopathies. Int. Ophthalmol..

[28] Daibert-Nido M., Patino B., Markowitz M., Markowitz S.N. (2019). Rehabilitation with biofeedback training in age-related macular degeneration for improving distance vision. Can. J. Ophthalmol..

[29] Sharma P., Tandon R., Kumar S., Anand S. (2000). Reduction of congenital nystagmus amplitude with auditory biofeedback. J. Am. Assoc. Pediatr. Ophthalmol. Strabismus.

[30] Daibert-Nido M. (2020). Biofeedback training for pediatric nystagmus: Improving visual functions and quality of life. Investig. Ophthalmol. Vis. Sci..

[31] Baker C.I., Peli E., Knouf N., Kanwisher N.G. (2005). Reorganization of visual processing in macular degeneration. J. Neurosci..

[32] Malvasi M., Compagno S., Segnalini A., Malvasi V.M., Pacella F., Turchetti P., Pacella E. (2024). Effectiveness of MP-3 microperimetric biofeedback fixation training for low vision rehabilitation in patients treated with corticosteroid IVT in retinal vein occlusions. Clin. Optom..

